# Interventions for cognitive frailty: developing a Delphi consensus with multidisciplinary and multisectoral experts

**DOI:** 10.3389/fnagi.2025.1541048

**Published:** 2025-06-04

**Authors:** Carol A. Holland, Nikolett Dravecz, Susan Broughton, Lynne A. Barker, Fidelia Bature, Charlotte Clarke, Isaac M. Danat, Sayani Das, Irundika H. K. Dias, Annabel Dawson, M. Dixon, Amanda Ellison, David Façal, Roland Finch, Christopher J. Gaffney, Alan Gow, Eirini Kelaiditi, Andrzej Klimczuk, Esperanza Navarro-Pardo, Pheobe Sharratt, Andrew Sixsmith, Claudia K. Suemoto, Lalu Suprawesta, Tamlyn Watermeyer, Sally Fowler Davis

**Affiliations:** ^1^Centre for Ageing Research (C4AR), Lancaster University, Lancaster, United Kingdom; ^2^Division of Health Research, Lancaster University, Lancaster, United Kingdom; ^3^Division of Biomedical and Life Sciences, Lancaster University, Lancaster, United Kingdom; ^4^Sheffield Institute of Social Science, College of Social Science and Art, Sheffield Hallam University, Sheffield, United Kingdom; ^5^Institute of Health Research, University of Bedfordshire, Luton, United Kingdom; ^6^Wolfson Institute for Health & Wellbeing, Durham University, Durham, United Kingdom; ^7^Sheffield Institute of Social Sciences, College of Social Sciences and Arts, Sheffield Hallam University, Sheffield, United Kingdom; ^8^Department of Social Work, Bar-Ilan University, Ramat Gan, Israel; ^9^Aston Medical School and Aston Research Centre for Health in Ageing, Aston University, Birmingham, United Kingdom; ^10^Lay Contributor, Newcastle, United Kingdom; ^11^Northern Health Science Alliance (NHSA), Manchester, United Kingdom; ^12^Department of Psychology, Durham University, Durham, United Kingdom; ^13^Department of Developmental and Educational Psychology, and Institute of Psychology, University of Santiago de Compostela, Santiago de Compostela, Spain; ^14^Lancaster Medical School, Lancaster University, Lancaster, United Kingdom; ^15^Department of Psychology, Heriot-Watt University, Edinburgh, United Kingdom; ^16^Faculty of Sport, Technology and Health Sciences, St Mary's University Twickenham, London, United Kingdom; ^17^SGH Warsaw School of Economics, Warsaw, Poland; ^18^Department of Developmental and Educational Psychology, University of Valencia, Valencia, Spain; ^19^Department of Gerontology, STAR Institute, Simon Fraser University, Vancouver, BC, Canada; ^20^Division of Geriatrics, Universidade de São Paulo, São Paulo, Brazil; ^21^Department of Health and Sport Education, Faculty of Sport Science and Public Health, Mandalika Education University, Mataram, Indonesia; ^22^Department of Psychology, Northumbria University, Newcastle upon Tyne,, United Kingdom; ^23^School of Allied Health and Social Care, Anglia Ruskin University, Cambridge, United Kingdom

**Keywords:** cognitive frailty (CF), intervention, Delphi study, expert consensus, multidisciplinary, multi-sectoral

## Abstract

**Introduction:**

The conjunction of physical frailty and cognitive impairment without dementia is described as Cognitive Frailty (CF). Indications that CF is potentially reversible have led to proposals that risk factors, symptoms or mechanisms of CF would be appropriate targets for interventions for prevention, delay or reversal. However, no study has brought experts together across sectors to determine targets, content or mode of interventions, and most resources on interventions are from the perspective of academic or clinical researchers only. This international Delphi consensus study brings together experts from academic and clinical research, lay people with lived experience of CF, informal carers, and professional care practitioners/clinicians.

**Methods:**

Three rounds of Delphi study were held to discern which factors and statements were agreed upon by the whole sample and which generated different views in those with differing expertise. A scoping review and Round 1 (29 participants) were used to gather initial statements. In Round 2, 58 people responded to statements and open text items, comprising 7 lab-based researchers, 27 researchers working with people, 14 people with lived experience or informal family carers, and 10 professional carers/clinicians. Percent agreement and qualitative responses were analyzed to provide a final set of statements which were checked by 38 respondents in Round 3.

**Results:**

Analysis of Round 2 quantitative data provided 74 statements on which there was at least 70% agreement and qualitative data produced a further 24 statements. These were combined to provide 90 statements for Round 3. There was Consensus for 89 of the statements. A few differences between the groups were observed at both stages.

**Discussion and conclusion:**

The consensus for statements associated with CF interventions provides a useful first step in defining health promotion activities and interventions. Given the prevalence and potential disability caused by CF in older populations, the consensus statements represent expert opinion that is inter-sectoral and will inform public health policies to support implementation of evidence-based prevention and intervention plans. This study is an important step toward changing current approaches, by including all stakeholders from the outset. Outcomes can be used to feed into co-creation of interventions for cognitive frailty.

## Introduction

Cognitive frailty (CF) is a condition defined as an age-related conjunction of physical frailty and cognitive impairment without dementia (Kelaiditi et al., 2013). Research on CF is growing, indicating, for example, that distinguishing the cognitive impairments and any neuropathological changes shown in CF from neurodegeneration associated with early dementia is essential (Bunce et al., 2019; Façal et al., 2019). This is important because CF is described as a state in which there is an increased risk for eventual dementia and loss of independent function, but also because it has been described as reversible, or potentially reversible (Ruan et al., 2020; Ruan et al., 2015). In the current absence of a cure for dementia, increasing prevention efforts is essential. That CF is a potentially reversible syndrome has led to the proposal that addressing risk factors, symptoms or mechanisms of either cognitive impairment or physical frailty, or importantly, the mechanisms associated with the link between the two, would be appropriate targets for intervention to prevent, delay or reverse it (see also Holland et al., 2024). Importantly, this highlights CF not just as a problem that may grow with increasing older populations, but as an important opportunity for healthy aging. However, despite the development of our understanding, CF remains poorly addressed as a target for intervention and perhaps poorly understood amongst the range of people with different kinds of expertise and experience in this area.

Physical frailty has been defined as (i) a physical phenotype (Fried et al., 2001) in which a frail state is the presence of three or more of five characteristics of physical weakness (slow gait speed, self-reported exhaustion, muscle weakness, low physical activity and unintended weight loss); and (ii) by an accumulation of deficits approach, or frailty index (Rockwood and Mitnitski, 2007). Both definitions relate to a recognized syndrome characterized as a state of increased vulnerability to adverse health outcomes when exposed to a stressor (Clegg et al., 2013). Underlying biological changes influence homeostatic mechanisms and absence of physiological resilience, including muscle wasting, metabolic deficits, cardiovascular disease, inflammatory symptoms and oxidative stress. The presence of both cognitive impairment and physical frailty may exacerbate vulnerability to adverse health outcomes, underscoring the need for integrated interventions targeting the unique mechanisms of cognitive frailty,

Cognitive Impairment No Dementia (CIND), the level of cognitive impairment at the focus of CF, has been defined in various ways, but the 2013 consensus exercise (Kelaiditi et al., 2013) suggested a Clinical Dementia Rating (CDR) of 0.5 (equivalent to “very mild” observed impairment). Other literature has emphasized an equivalence with the Mild Cognitive Impairment (MCI) range on global tests of cognition such as the Mini Mental State Exam (MMSE; Folstein et al., 1975) or Montreal Cognitive Assessment (MOCA, Nasreddine et al., 2005), or a combination of tests of specific cognitive domains such as verbal memory or executive function (e.g., see Façal et al. (2019) for a review) and others have emphasized subjective cognitive decline, particularly for a reversible level of CF (Ruan et al., 2015). Nevertheless, the key defining feature of CF, is that the cognitive impairment is in conjunction with physical frailty.

Development of interventions for physical frailty (e.g., Apostolo et al., 2018) and interventions for mild cognitive decline (e.g., Levy et al., 2022) have received significant attention. To date and to our knowledge, there are no agreed pathways for intervention for CF. Several reviews have been published examining interventions for CF, focusing on different mixes of intervention targets: physical exercise interventions (Li et al., 2022); a mixture of cognitive training, nutrition education, behavioral intervention, mind–body intervention, psychosocial support, and virtual reality but all with a component of physical activity (Tam et al., 2022); physical activity, nutrition or multidomain interventions (Zheng et al., 2022); nutrition only, with a focus on anti-oxidants (Gomez-Gomez and Zapico, 2019); and multidimensional and potentially personalized interventions (Sugimoto et al., 2022). However, no study has sought cross-disciplinary expertise to discuss the potential for interventions or brought together expertise and opinions on what might work in terms of targeted outcomes and structures of interventions. Importantly, published resources on interventions are primarily from the perspective of academic or clinical researchers, and lack the perspective of those with lived experience, e.g., informal carers and people with CF.

The objective of this study was to conduct an international Delphi consensus study bringing together experts from a range of academic and clinical research backgrounds, while also ensuring the involvement of experts by experience: lay people with lived experience of CF themselves; informal (family) carers; and professional health and social care practitioners. Hsu and Sandford (2007) defined a Delphi consensus as “a group communication process which aims to achieve a convergence of opinion on a specific real-world issue.” Delbecq et al. (1975) described the different potential objectives of a Delphi process which include: exploring or exposing underlying assumptions or information that may lead to different judgments; seeking information that may generate consensus amongst participants; and finding linkages between different judgments in a topic that spans a range of disciplines. Delphi techniques are considered to provide the lowest level of evidence for making causal inferences, i.e., subordinate to meta-analyses and intervention studies (Liu, 2022) but are appropriate particularly for consumer review in guideline development and in relation to complex issues where knowledge is uncertain (Linstone and Turoff, 2002). Importantly, the process is iterative in which a series of questionnaires can be used so that each member can contribute independently, and anonymously, and each round is a controlled feedback process of the information gained in the previous round, reducing the possibility of bias or undue influence of some experts, especially where there is a perceived power imbalance.

Methodological sources suggest that a mix of experts provides clearer face validity for any consensus achieved (e.g., Nair et al., 2011), particularly where the motivation of end users may be a key component of potential outcomes – the interventions in this case. This mix of experts was chosen to ensure that consensus on what might work in terms of scientific evidence was combined with what might work for people who may be the recipients, beneficiaries and those who may deliver the interventions.

The use of ‘experts’ is fundamental to the reliability of the outcome and rigour is associated with selection of the experts (Baker et al., 2006). Nevertheless, it was also recognized that understanding in some areas, notably biological, pharmacological, medical and neuroscience mechanisms, may be less well-developed within the range of experts included. Therefore, in addition to seeking overall consensus across the participants as in a traditional Delphi study, we also aimed to determine whether there were any significant differences between the different groups of participants.

Thus, the aims of this Delphi study were to:

- Develop consensus among experts across disciplines and sectors, including people with lived experience, in relation to understanding of cognitive frailty and appropriate interventions for it.
- Examine differences in consensus between groups with different disciplinary backgrounds and lived experience.

## Methods

The process included three rounds to achieve expert consensus on sub-components of interventions, as follows:

- Understanding of what CF is, whether it is preventable, could be delayed, or could be reversible;
- possible mechanisms or approaches for interventions, and target foci for interventions;
- any restrictions on for whom interventions for CF may or may not be relevant;
- screening for CF;
- factors that may affect the feasibility or likelihood of adherence to interventions, including effects of infrastructure, accessibility or personal circumstances on feasibility of interventions;
- potential targeted primary and secondary outcomes;
- factors in the design of interventions that may influence effectiveness;
- and any other design factors.

Following an initial literature search activity as part of a scoping review, (Holland et al., 2024), three Delphi rounds were held. Ethical approval was granted in advance by Lancaster University Faculty of Health and Medicine Ethical Committee (REF FHM-2022-1092-RECR-1) and updated using amendments as the survey was developed for each round (FHM-2023-1092-SA-1).

### Round 1

The first round was completed as an event at an online Cognitive Frailty Interdisciplinary Network (CFIN) conference in September, 2023. Open questions based on the scoping work (Holland et al., 2024) were presented on a shared electronic notepad (“Padlet”) which participants discussed in small groups and contributed text answers within their groups. Twenty-nine experts who had mostly presented their own work in the area of cognitive frailty at the conference, including experts in psychology, neuropsychology, sociology, basic lab based biological sciences and physiology took part in the exercise, alongside two lay members of the network external advisory group (people with lived experience or with professional or informal carer expertise). All those who wanted to participate were given a participant information sheet and consent form which ensured they were aware that responses would be anonymous, but contact details would be separately retained to invite them to the second round.

### Round 2

The second round consisted of an on-line survey based on responses to Round 1 distilled into a set of 127 items suitable for rating as agree/disagree, or ranking, according to the question, plus open text boxes. The items were converted into a questionnaire which was developed as an online Qualtrics questionnaire and reviewed by the chair of the advisory group. The list of items can be seen in the Supplementary Materials 1. Agree/disagree items were to be answered using a 5-point Likert scale, from strongly agree to strongly disagree. Ranked items consisted of a series of elements to be ranked in order of importance. The set of items included all contributions from Round 1 with none excluded at this stage.

To extend the participant group of experts the following actions were taken:

- All members of CFIN who had not taken part in Round 1 were invited;
- Further published experts were invited;
- Members of the VOICE^1^ network of older citizens were invited specifically to increase the numbers of people with lived experience of CF themselves or experience of caring for people with CF.

Fifty-eight participants took part in Round 2, with 46 completing all questions. Partial responses were included in the analysis and numbers responding to each item are given in the analysis below. All interested individuals were provided with participant information sheets and consent forms via email. Participants were then sent the link to the questionnaire and asked to complete it online. At the end of the questionnaire, participants were asked to provide contact details so they could be involved in a further round and/or in writing or editing this report.

### Analysis for Round 2

Based on a previous Delphi process in this area (Sezgin et al., 2022) and on recommendations from Nair et al. (2011) an agreement level of 70% was applied to agree/disagree questions. That is, responses rated “somewhat agree” or “strongly agree,” or, strongly disagree” or “somewhat disagree” by ≥70% of participants were accepted unless there was a sizeable opposite proportion, i.e., “strongly disagree” and “somewhat disagree” or “strongly agree” and “somewhat agree” by more than 15%. Participants who responded that any question was outside of their expertise were excluded from the percentage calculations for that item. For ranking questions, mean ranks for each item were calculated and a median overall mean rank or above was determined as demonstrating consensus. Friedman’s non-parametric test for ranked data was applied to test the null hypothesis of no difference in mean ranks across the items (that is, the hypothesis of no consensus on higher and lower ranks of items).

Following analyses of respondents as a whole, respondents were separated into groups in terms of their different types of work or association to CF based on their own descriptions of their expertise or interest in CF: laboratory-based researchers; researchers who worked directly with older people with CF or data relating to them; health and social care practitioners who worked with people with CF; and people with lived experience either as informal (usually family) carers or people self-declaring as experiencing CF themselves. Grouping participants by expertise allowed for an analysis of consensus differences based on professional or personal perspectives, providing insights into how lived experiences and disciplinary backgrounds influence cognitive frailty intervention priorities. Independent samples Kruskal-Wallis tests were used to examine whether there were any differences in the distribution of ranks for each intervention between the groups, with Bonferroni adjustments made for multiple comparisons.

Qualitative responses (open text boxes) were subjected to thematic analysis (Brady, 2015) that identified additional priorities, gaps in understanding and additional issues of importance (Shang, 2023) and put them into the format of further statements. This enabled a ‘sense check’ for the development of further statements, with qualitative outcomes synthesized with the quantitative item outcomes and formulated into a number of statements that could be incorporated into appropriate sections of Round 3 of the survey.

### Round 3

All statements that had achieved consensus or had been added from the qualitative analysis were formatted into items that could be answered using the strongly agree to strongly disagree scale outlined above (that is, previously highly ranked items were re-formatted into agree-disagree statements). This was emailed to all participants from Round 2 who had agreed to be contacted again.

For the third round, the group of experts were again divided into four groups as in Round 2, but with people asked to indicate the group themselves as opposed to the researchers allocating based on participant descriptions. In addition, country, perspective (e.g., academic researcher), education, role, discipline, was also requested while still allowing the respondent to remain anonymous.

### Analysis plan for Round 3

The same ≥70% agreement and ≤15% disagreement criteria for consensus were applied, excluding those who noted the question was outside their expertise. Analyses were conducted with the whole sample, as well as with respondents grouped by expertise.

## Results

Initial contributions to the Padlet in Round 1 were synthesized into items for the questionnaire in Round 2.

### Round 2

#### Participants

**Figure 1:**
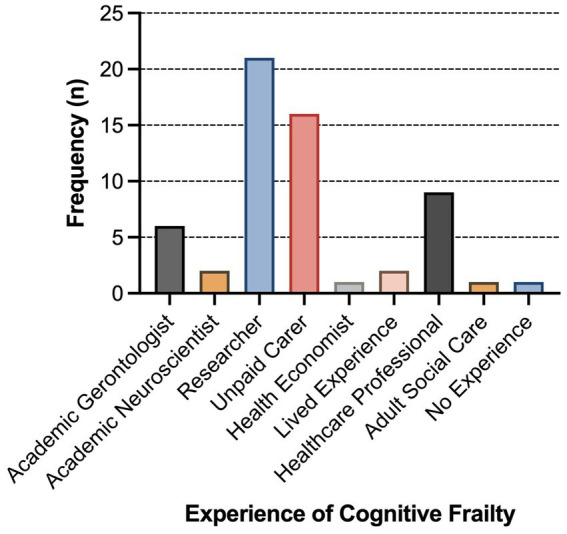
Frequency of categories of self-identified expertise (Figure preparation was conducted using GraphPad Prism 10 (GraphPad Software 2365 Northside Dr. Suite 560 San Diego, CA 92108)).

Fifty-eight people completed the survey in Round 2. The first question asked about their experience with CF. Several identified themselves in more than one category (e.g., having cared for a family member with CF but also experiencing it themselves; being a healthcare professional (clinician) and a researcher; being a researcher but also caring for a family member). Figure 1 shows all these self-identifications and so the total frequency is greater than the number of people. The most frequent types of experience identified were as researchers and as unpaid, or family, carers. One respondent said they had no experience or expertise of any nature with CF and were excluded from the analysis.

##### Overall consensus regarding the statements

Statements were examined in relation to the level of agreement observed, with 58 respondents answering most questions. Percentages are for agreement unless otherwise stated, and based on 58 respondents except where stated (missing responses or where participants did not answer a particular item because they did not perceive they had the expertise for that item). There were 7 Academic lab scientists/biologists, 27 Academics who worked with people, 14 people with lived experience and carers, and 10 professional carers (Clinicians/social care). Items where consensus was achieved according to our criteria are highlighted in **bold** in their respective results table. Extracted qualitative statements are appended in each table as relating to the items in relation to which they had been given.

##### Section 1: understanding and thinking about cognitive frailty

In the statements in Table 1, there was only clear agreement only for items 1.1 and 1.5. Participants agreed that “Many researchers and clinicians are not clear what is meant by cognitive frailty” and that “Negative attitudes toward aging are what needs changing.” For Item 1.2, “Cognitive frailty is just a part of the aging process,” there was more disagreement than agreement, but no consensus by our criteria.

**Table 1:** Understanding and thinking about cognitive frailty: percentage agreement.

Group	Academic lab scientist/biologist	Academic working with people	People with lived experience and unpaid carers	Professional carers (clinicians, social care)	Overall agreement/disagreement, *n* = 58
**1.1 Many researchers and clinicians are not clear what is meant by cognitive frailty**	100	92.31	60.00	80.00	84%
1.2 Cognitive frailty is just a part of the Aging process	42.86	84.60 (disagree)	53.40	30.00	62% (disagree)
1.3 Cognitive frailty is a physiological condition	100	37.00	50.00	80.00	55.7%
1.4 Cognitive frailty is experiential/subjective	71.43 (disagree)	46.15	61.54	30.00	43.4%
**1.5 Negative attitudes toward aging are what need changing**	71.43	80.77	85.71	80.00	80%
1.6 “Aging” is a social construct	85.72 (disagree)	46.15	50.00	70.00	49.1%
1.7 Is it a help/hindrance to normalize cognitive frailty as an expected part of aging?	57% help/43% hindrance	33% help/67%hindrance	50% help/50% hindrance	30% help/70% hindrance	41% help/59% hindrance
**Qualitative statements from the open text boxes in Section 1**	*Whilst normal aging may contribute to cognitive frailty, it is unhelpful to assume that cognitive frailty is an expected part of aging.*				

Items where consensus was achieved according to our criteria are highlighted in bold.

There was one further question in this section: “Item1.7: Is it a help or a hindrance to normalize cognitive frailty as an expected part of aging?” Of 58 responses, 58.62% responded that it was a hindrance. That is, there was no overall agreement.

Table 1 also shows the percentage agreement (or disagreement) with the statements as they varied within the different expertise groups. Although there was agreement on some issues, important differences emerged in relation to the statement on cognitive frailty being a physiological condition, with biologists and clinicians agreeing that it was, but other groups showing less consensus. Biologists also disagreed that aging was a social construct, whereas other groups, to some extent, agreed that it was. Academic researchers working with older people disagreed that CF is just part of the aging process, with other groups showing no consensus. Finally, there was a broad agreement amongst researchers working with people, and professional carers (such as clinicians) that it was a hindrance to normalize CF as an expected part of aging, while there was no consensus in other groups. This was further illustrated by the statement from the qualitative data, that *Whilst normal aging may contribute to cognitive frailty, it is unhelpful to assume that cognitive frailty is an expected part of aging.*

##### Section 2: preventability of cognitive frailty

There was clear overall consensus on all the items in Section 2, see Table 2.

**Table 2:** Preventability, delay and reversibility of cognitive frailty (52–55 responses).

Group	Academic lab scientist/biologist	Academic working with people	People with lived experience and unpaid carers	Professional carers (clinicians, social care)	Overall % agree/disagree
**2.1 Cognitive frailty can be prevented**	100	100	92.86	100	98.2%
**2.2 Evidence suggests that cognitive frailty may be delayed or reduced, but we do not know yet the extent to which it can be prevented**	100	100	92.86	90.00	96.4%
**2.3 We know some of the risk factors or predictors for cognitive frailty so that means it can be addressed**	85.71	96.30	92.86	90.00	92.73%
**2.4 We know some of the biological mechanisms that underlie cognitive frailty so they could be targeted to prevent cognitive frailty**	100	77.78	77.78	100.00	88.4%
**2.5 Social and psychological factors that may act as risk factors would involve long term changes and may be harder to change**	66.67	87.50	71.43	90.00	81.5%
**2.6 Policy changes are needed to influence psychosocial/ socioeconomic factors**	83.33	92.59	80.00	88.89	91.5%
2.7 What is the most important factor when it comes to the prevention of Cognitive Frailty?	14.29	0.00	7.14	0.00	3.51%
Psychosocial factors	0.00	7.41	7.14	20.00	10.53%
Socioeconomic environment	28.57	3.70	0.00	0.00	7.02%
Biomedical factors	57.14	88.89	85.71	80.00	78.95%
All 3 are equally important					
**3.1 Cognitive frailty can be delayed**	100.00	100.00	100.00	100.00	100%
**3.2 Delaying cognitive frailty depends on behavior**	83.55	88.00	78.57	70.00	82.7%
3.3 Delaying cognitive frailty depends on genetics.	83.33	72.73	25.43	50.00	57.8%
**3.4 Delaying cognitive frailty depends on identifying risk factors early, when risk factors or symptoms are first seen in a person**	83.33	92.00	92.31	80.00	90.2%
3.5 The risk factors for cognitive frailty act individually	16.67	44.00	30.77	40.00	37%
**3.6 The risk factors for cognitive frailty interact in complex ways**	85.71	91.30	66.67	85.60	86.4%
**3.7 There are key factors that would have significant effects on other factors if we could target interventions on them**	57.14	86.50	50.00	88.89	78.7%
**3.8 Cognitive frailty is often not identified until someone has a serious illness/injury and comes into contact with health care practitioners**	100.00	78.26	71.43	90.00	81.3%
**3.9 Early assessment/screening of older people would help identify factors for earlier intervention**	100.00	96.00	100.00	90.00	95.8%
**Qualitative statements from the open text boxes in Section 3**	*It is likely that with preventative interventions cognitive frailty can be delayed or reduced.* *Lifestyle factors, diet and exercise, and hereditary factors including genomic profile (i.e., APOE ε4) may influence the likelihood of becoming cognitively frail.* *Psychosocial, socioeconomic, environmental and biomedical methods are potential interventions to reduce or reverse cognitive frailty.*				
4.1 Cognitive frailty can be reversed	83.33	75.00	53.85	80.00	72%*
**4.2 Cognitive frailty might not be reversible, but it could be improved**.	100.00	79.17	91.67	80.00	87.8%
**4.3 Examination of reversal of cognitive frailty in the literature is very rare**	100.00	72.73	57.14	87.50	74.4%
**4.4 We do not know the best timing (e.g., in terms of severity of cognitive frailty or age of person) for interventions to enable reversal**	100.00	65.22	44.44	70.00	72.1%
4.5 Reversibility has a threshold in terms of severity of the cognitive frailty	60.00	72.73	40.00	80.00	68.4%
4.6 Reversibility may be possible but only for a period	80.00	71.43	44.44	50.00	56.1%
**Qualitative Statements from open text boxes in Section 4**	*Cellular inflammation may be a biological mechanism associated with cognitive frailty.* *Social isolation and the consequent loss of participation in community may exacerbate cognitive frailty.* *Air pollution affects brain health especially for those with pre-existing vulnerability leading to cognitive frailty.* *Reversal of cognitive frailty is associated with positive attitudes and expectations of aging and the consequent resilience to age related stress.*				

Items where consensus was achieved according to our criteria are highlighted in bold.

When these data were considered in terms of the four expertise groups, there were similar levels of agreement, except for academic laboratory scientists who did not show the required 70% consensus on items 2.5 (consensus was 66.67%) or 2.7. For 2.7, over 80% of respondents in all other groups agreed that all three factors (Psychosocial, Socioeconomic environment, Biomedical factors) were equally important in the prevention of CF, whereas only 57.14% of the laboratory scientists (biologists) endorsed that statement.

##### Section 3: whether cognitive frailty could be delayed

As can be seen in Table 2, all statements achieved >70% agreement except items 3.3 “Delaying cognitive frailty depends on genetics” (notably 13.46% of respondents did not feel they had the expertise to answer), and 3.5 “The risk factors for cognitive frailty act individually” where 42% of respondents disagreed with this statement and 37% agreed with it, showing differing opinions. The two kinds of academic researchers agreed that delaying CF depends on genetics (Item 3.3), whereas people with lived experience and unpaid carers, and professional carers/clinicians, showed no consensus on this topic. The qualitative statements emphasized that interventions and lifestyle factors influence the likelihood of becoming cognitively frail, but also acknowledged the role of genetic factors and suggested a range of approaches for intervention, namely psychosocial, socioeconomic, environmental and biomedical.

##### Section 4: whether cognitive frailty is reversible

As can be seen in Table 2, three of the six statements showed consensus. Items 4.5 “Reversibility has a threshold in terms of severity of the cognitive frailty” and 4.6 “Reversibility may be possible but only for a period” showed differing opinions. Item 4.1 “Cognitive frailty can be reversed” showed >70% agreement, but also 16% disagreement*, and so by our criteria, is not accepted as showing consensus. It is worth noting that this was an area where a number of people felt they did not have the expertise to respond to the statement, notably items 4.3 to 4.6. Of those who did not say that and so were included in the analysis, people with lived experience and carers showed the lowest consensus, notably for Item 4.1. It is also worth noting that the highest consensus was among lab-based scientists/biologists (who are among those who would be expected to have access to empirical evidence to back up this statement), and professional carers, who may see reversal of cognitive frailty in practice. The qualitative statements focused on potential mechanisms, such as cellular inflammation, social isolation and the consequent loss of participation in community, and air pollution. Finally, one comment suggested that reversal of CF is associated with positive attitudes and expectations of aging and consequent resilience to age related stress.

##### Section 5: intervention approaches and mechanisms

This section focused on the possible mechanisms of interventions for cognitive frailty.

There was strong agreement for all items except 5.13, where 20.4% of the respondents said the question on intervening with biological mechanisms was outside of their expertise, and 22.45% said they neither agreed nor disagreed. This suggests biological interventions are relatively unknown to participants. Even so, more people agreed than disagreed overall. Inspection of the percentage agreement for the different groups showed that only the academic laboratory-based researchers showed consensus for this item. The professional carers/clinicians group also showed no consensus for items 5.1, 5.2 and 5.16, although all other groups did. Results can be seen in Table 3. The statements extracted from the qualitative data gave suggestions for interventions or their implementation, or information on those currently being trialed, recommending sustained engagement with the target population or individual and on community facilities. A final statement emphasized the importance of addressing ageism by positive-age-belief interventions which could influence the progression of CF.

**Table 3:** Approaches to interventions for cognitive frailty, Section 5 (50 responded to Questions 5.1 to 5.12, and 49 answered 5.13 to 5.16).

Group	Academic Lab scientist/biologist	Academic working with people	People with lived experience and unpaid carers	Professional carers (clinicians, social care)	Overall % agree/disagree
**5.1 Translational pre-clinical studies are important**	100.00	85.71	80.00	66.67	81%
**5.2 Interdisciplinary working to test interventions in different models and systems (including cell culture and animal models from invertebrate to vertebrate) will help to improve likelihood that they are clinically relevant**	100.00	78.26	83.33	60.00	78.7%
**5.3 One size does not fit all - interventions need to be personally relevant (e.g., acceptable in different cultures)**	100.00	95.83	85.71	80.00	92%
**5.4 One size does not fit all–interventions need to be personalized to needs (e.g., to address specific health needs of the person, as opposed to general for the population)**	100.00	100	85.71	90.00	94%
**5.5 Quality of life should be a focus: improving this could have an impact on decline in function (e.g., engaging in activities, reducing loneliness).**	100.00	100.00	100.00	100.00	100%
**5.6 The role of the individual is important, rather than just a focus on biological or environmental mechanisms**	66.67	95.83	100.00	90.00	91.8%
**5.7 Intervention targets may be different depending on whether you are targeting prevention, delay or reversal**	100.00	91.67	83.33	80.00	89.6%
**5.8 For prevention, lifestyles should be targeted**	83.34	95.83	92.31	90.00	91.8%
**5.9 The main effective target would be health behaviors including physical activity**	83.34	86.96	92.86	70.00	85.7%
**5.10 Understanding the mechanisms should lead to a focus on multi-domain intervention (a mechanism could be defined as how a risk factor might have its effect)**	83.34	100.00	90.00	90.00	93.5%
**5.11 If we consider that all aspects (biological, psychosocial, socioeconomic, lifestyle, aging, etc.) are important, then looking for common mechanisms across them may inform changes that we can make to prevent or treat cognitive frailty**	66.67	91.30	92.86	90.00	87.5
**5.12 Biological mechanisms that are affected by health behaviors, psychosocial and socioeconomic factors should be a focus**	100.00	86.96	81.82	87.50	86.4%
5.13 Intervening on more direct biological mechanisms may be quicker	83.33	55.00	55.56	37.50	56.4%
**5.14 For some people there may be a need for a fast-acting intervention**	100.00	90.91	76.92	80.00	87.2%
**5.15 For others, a longer-term intervention might be useful (e.g., long term lifestyle modification)**	100.00	100.00	76.92	90.00	91.3%
**5.16 Short burst versus longer-term interventions may be suitable for different people, but also for addressing different mechanisms**	100.00	90.91	70.00	66.67	83.7%
**Qualitative statements from open text boxes in Section 5**	*Lifestyle interventions (improved nutrition and physical activity) are currently being trialed and may enable reversal of cognitive frailty.* *Lifestyle interventions depend on effective implementation and sustained engagement with the target population or individual and on community facilities.* *Exposure to ageism is mitigated by positive-age-belief interventions and may influence the incidence of cognitive frailty.*				

Items where consensus was achieved according to our criteria are highlighted in bold.

##### Section 6: intervention targets

This section focused on designing interventions for cognitive frailty. Participants were asked to rank eleven proposed intervention targets with 1 being the most important and 11 the least. Forty-six people responded to this question.

Using Friedman’s non-parametric test for ranked data, there was a significant difference in the overall ranking between items [Chi-squared = 138.07 (df = 10), *p* < 0.001]. That is, there was agreement in the ranked order of the intervention factors across respondents, although there was still some variance. Physical activity + diet + probiotics was ranked the most important, followed by “Targeting isolation and loneliness/inclusive environments“, “Strength related exercise, not just cardiovascular,” and “Whole patient is important, e.g., caring for other morbidities such as arthritis.” Addressing menopausal effects on muscle was ranked as the least useful, as seen in Table 4.

**Table 4:** Mean Ranking for Intervention targets in Section 6.

Intervention characteristic	Academic lab scientist/biologist	Academic working with people	People with lived experience and unpaid carers	Professional carers (clinicians, social care)	Mean rank Overall	SD
**Physical activity + diet + probiotics**	1.40	3.52	3.31	2.80	3.17	2.59
**Targeting isolation and loneliness/inclusive environments**	5.60	4.16	3.15	3.20	3.94	2.54
**Strength related exercise, not just cardiovascular**	4.00	4.56	5.23	6.00	4.83	3.01
**Whole patient is important, e.g., caring for other morbidities such as arthritis**	6.00	4.92	4.00	6.40	4.94	3.18
Increasing social engagement	5.80	5.40	4.23	5.00	5.08	2.44
Mental wellbeing and mental health interventions as appropriate	7.60	5.52	5.00	6.20	5.67	2.79
Occupational factors, intellectual stimulation	7.80	7.00	6.00	7.80	6.90	2.54
Treatment and prevention focused on vascular factors	7.20	6.72	8.92	5.60	7.25	2.89
Addressing oxidative stress	8.20	7.24	8.62	5.80	7.56	2.50
Drug based interventions for inflammaging	5.20	8.44	8.15	8.20	8.00	2.58
Addressing menopausal effects on muscle	7.20	8.52	9.38	9.00	8.67	2.51
**Qualitative Statements from open text boxes in Section 6**	*Therapeutics must be developed to address dysregulation across multiple cellular processes including genetic alterations, nutrient and lipid metabolism, and pro-inflammatory proteins.* *Person centered assessment and rehabilitation interventions may enable functional improvement related to cognitive frailty.* *Wellbeing interventions for older people, based on inclusivity and health promotion are likely to improve population level outcomes, particularly for more socially deprived groups.* *Preferred metrics by which to measure improvements/reversal of cognitive frailty include improved mobility and cognition and reduced frailty score or reversal to non-cognitive frailty profile* *Other outcomes may include social or economic independence, relative to cultural values and perception of elders at community level*.					

Items where consensus was achieved according to our criteria are highlighted in bold.

In terms of a decision on which statements achieved consensus as important, we used a cut-off of the rank below the median (5.08). An independent samples Kruskal-Wallis test examined whether there were any differences in the distribution of ranks for each intervention across the groups. There were no overall effects (*p* > 0.05). In individual pairwise comparisons, using Bonferroni adjustment for multiple comparisons, again, there were no significant differences.

The qualitative statements in this section were diverse in terms of suggested intervention targets, ranging from biomedical therapeutics to person centered assessment and rehabilitation and wellbeing interventions based on inclusivity and health, particularly for more socially deprived groups. Further statements endorsed outcomes such as improved mobility and cognition and reduced frailty score or reversal to non-cognitive frailty profile but also suggested social or economic independence, relative to cultural values and perception of elders at community level.

##### Section 7: possible restrictions on for whom interventions for cognitive frailty might be suitable

As seen in Table 5, only one item, 7.2 “Agency and involvement of the person is vital in agreeing on the intervention” achieved consensus. Item 7.6 “Lifestyle interventions are difficult to implement if individuals have limited social support” gained more than 70% agreement, but also 18.75% disagreement, with more disagreement in the academic lab scientist group. Only the Professional carers/clinicians showed consensus on item 7.1 referring to possible restrictions for whom interventions might be suitable, and this group joined those with lived experience in showing consensus over the difficulty of implementing lifestyle interventions if people have mobility issues.

**Table 5:** Factors that may affect for whom interventions are suitable (Section 7), on factors related to screening (Section 8) (*N* = 48 for Section 7, *N* = 48 for Items 8.1 and 8.2 and 47 for item 8.3).

Factors affecting suitability and screening	Academic Lab scientist/biologist	Academic working with people	People with lived experience and unpaid carers	Professional carers (clinicians, social care)	Overall % agree/disagree
7.1 There would be restrictions on who interventions for cognitive frailty might be suitable for	50.00	61.90	38.46	77.78	52.3%
**7.2 Agency and involvement of the person is vital in agreeing on the intervention**	100.00	100.00	92.86	100.00	95.9%
7.3 Lifestyle interventions are difficult to implement if individuals have mobility issues	66.67	63.64	71.43	70.00	64.6%
7.4 Lifestyle interventions are difficult to implement if individuals have dietary restrictions	50.00	45.45	42.86	60.00	45.8%
7.5 Lifestyle interventions are difficult to implement if individuals have distinct cultural practices	33.34	54.54	50.00	50.00	47.9%
7.6 Lifestyle interventions are difficult to implement if individuals have limited social support	66.67	77.27	78.57	80.00	75%
**8.1 Screening for cognitive frailty is essential**	83.33	86.36	78.57	90.00	85.1%
**8.2 High risk people should be identified at midlife**	83.33	90.91	71.43	90.00	85.1%
8.3 Screening as part of interventions: Confounding factors can impact the outcomes of interventions - screening for some of these may help to remove their influence in trials, but this then potentially excludes many people and could become highly restrictive	66.67	71.43	57.14	50.00	65%
**Qualitative statements from open text boxes in Section 8**	*Risk factors for cognitive frailty interact in complex ways but earlier screening bio markers can be identified.* *Effective strategies for reducing cognitive frailty at population level (ie large sample size/recruitment) suggest the need for cohort studies with multi-factorial screening* *Traditional screening methods for cognitive frailty may need to be enhanced with broader understanding social determinants of health,* i.e.*, access to community assets, and other environmental factors.*				

Items where consensus was achieved according to our criteria are highlighted in bold.

##### Section 8: screening for cognitive frailty

As seen in Table 5, there was consensus for the items 8.1 “Screening for cognitive frailty is essential” and 8.2 “High risk people should be identified at midlife.” For item 8.3, the more complex statement around confounding factors, 8 people responded that it was outside of their expertise and 23.4% neither agreed nor disagreed. Within the expertise groups, academics working with people did show consensus agreement. The extracted qualitative statements provide some more detail to the positive consensus on the importance of screening, in terms of suggesting that early screening biomarkers could be identified, as well as suggesting that multi-factorial screening and a broader understanding of social and environmental determinants of health should be incorporated into screening and future cohort studies.

##### Section 9: feasibility of interventions for cognitive frailty

Item 9.1 asked respondents to rank eight factors that may affect the feasibility or likelihood of adherence for people in relation to interventions for CF, with 1 being the most important.

The Friedman’s test showed a significant overall difference in the ranking between the items [Chi-Squared = 139.53 (df = 7), *p* < 0.001], confirming agreement across respondents in the ranked order of the intervention factors that may restrict or affect the feasibility or likelihood of adherence, e.g., including accessibility, affordability, acceptability, and health literacy, although there was still some variance. As illustrated in Table 6, accessibility was ranked the most important, and ethnicity as the least important. In terms of a decision on which statements were achieving consensus as important, a cut-off of a rank below the median (4.68) was used.

**Table 6:** Ranking of factors affecting feasibility of interventions (*N* = 48, Sections 9.1, 9.3, and 9.5).

Intervention characteristic	Academic lab scientist/biologist (*n* = 6)	Academic working with people (*n* = 23)	People with lived experience and unpaid carers (*n* = 13)	Professional carers (clinicians, social care) (*n* = 6)	Mean rank	SD
Section 9.1 Ranking of factors affecting feasibility of interventions (*N* = 48)						
**Accessibility**	2.17	2.30	1.92	2.00	2.14	1.07
**Affordability**	2.50	2.96	3.31	2.17	2.89	2.12
**Acceptability**	3.33	3.39	3.31	4.83	3.52	1.75
**Health Literacy**	3.17	4.52	5.00	3.50	4.35	2.38
Accessibility of Information and Consent	5.50	5.09	4.69	4.83	5.01	1.88
Culture	5.17	5.31	5.17	5.33	5.33	1.43
Geography	7.17	5.74	5.85	6.50	6.04	1.91
Ethnicity	7.00	6.65	6.62	6.83	6.72	1.17
Section 9.3 Ranking of structural factors that may affect feasibility of interventions (*N* = 47)						
**Affordable/free access to classes or leisure centers**	2.83	3.52	4.31	3.80	3.68	1.76
**Access to cognitive frailty screening**	4.83	4.74	3.38	4.40	4.36	3.23
**Physically accessible environments**	5.33	4.09	3.77	6.80	4.46	2.40
**Accessible healthy foods**	3.3	4.52	5.08	4.40	4.51	2.18
Socially accessible environments	5.17	4.91	4.31	3.20	4.60	2.52
Transport to activity venues	6.50	4.70	5.08	3.40	4.89	2.33
Access to clinically supported interventions where indicated	4.83	6.04	5.23	6.20	5.69	2.71
Support to use technologically based interventions	6.33	6.48	5.69	7.00	6.30	1.84
Cycle lanes, safe walking and running paths	5.83	6.00	8.00	5.80	6.51	2.69
**Section 9.5 Ranking of factors relating to personal circumstances that may affect interventions, *N* = 47**						
**Affordability**	3.67	3.48	3.85	3.60	3.63	2.24
**Time factors, e.g., related to occupation or care responsibilities**	3.17	4.17	3.00	5.60	3.87	2.31
**Personal attitudes and beliefs (e.g., about the effectiveness of an intervention)**	4.33	3.96	3.31	4.60	3.89	2.59
**Mobility**	6.83	4.65	4.00	5.00	4.80	2.28
Understanding/health literacy	4.33	5.13	6.00	3.00	5.04	2.66
Own health beliefs	4.67	5.14	6.69	4.00	5.38	2.94
Personal goals	5.33	5.70	6.54	4.80	5.79	2.38
Family and Friends influences	6.33	6.04	5.54	7.00	6.04	1.98
Energy Levels	6.33	6.70	6.08	7.40	6.55	2.17
**Qualitative statements for Section 9**	*There is a preference for fully personalized interventions that address multi-factorial causes of cognitive frailty (supported by a decisional flowchart, or by a multidisciplinary team for instance).* *Co-design of cognitive frailty interventions is indicated for the purpose of identifying barriers to acceptability, accessibility and affordability whilst recognizing the need for scalability and transferability to different health economies.* *Preventative strategies are likely to involve environmental improvements (i.e., reducing air pollution) and inclusive community planning (i.e., active aging strategies)*					

Items where consensus was achieved according to our criteria are highlighted in bold.

Using the independent samples Kruskal-Wallis test, the distribution of ranks for each intervention across the groups was examined. There were no overall effects (*p* > 0.05). In individual pairwise comparisons between the groups, there were also no significant differences.

Question 9.3 asked participants to rank structural factors that may affect feasibility of interventions, where 1 means the most important. Forty-seven respondents completed the ranking, illustrated in Table 6:

Friedman’s test showed a significant overall difference in the ranking between the items [Chi-Squared = 45.76 (df = 8), *p* < 0.001]. That is, there was agreement in the ranked order of the structural factors that may restrict or affect the feasibility of involvement for respondents, although there was still some variance. Affordability or free access to classes or leisure centers was ranked as the most important, and cycle lanes, safe walking and running paths as least important. In terms of a decision on which statements achieved consensus as being important, the median cut-off was 4.60.

The extracted statements from the qualitative data (see Table 6) emphasized the need for personalized but multifactorial interventions, and the important role of co-design, scalability and transfer to different health economies.

The Kruskal-Wallis test to examine the distribution of ranks for each item across the groups found no overall effects (*p* > 0.05). In individual pairwise comparisons, there were no significant differences between the different groups of experts.

Item 9.5 asked participants to nine rank factors related to personal circumstances that may affect feasibility, with 1 being the most important. Friedman’s test showed a significant overall difference in the ranking between the items [Chi-Squared = 54.45 (df = 8), *p* < 0.001] demonstrating that there was agreement in the ranked order of the personal circumstance factors that may affect the individual feasibility of interventions, as illustrated in Table 6. Affordability was the most important personal factor, and personal energy levels seen as the least important. In terms of a decision on which statements achieved consensus as important, the median cut-off was 5.04.

The independent samples Kruskal-Wallis test showed no overall effects (*p* > 0.05) illustrating that the distribution of ranks for each intervention did not differ by group. In individual pairwise comparisons, again there were no significant differences between expert groups. The statements from the qualitive data (e.g., from questions 9.2 and 9.4, see Table 6) suggested that preventive strategies are likely to include environmental and inclusive community strategies, as well as focusing on personally focused strategies.

##### Section 10: primary and secondary outcomes of interventions for frailty

###### Primary outcomes

Using Friedman’s non-parametric test for ranked data, a significant overall difference in the ranking between the items was found (Chi-Squared = 23.30, df = 4, *p* < 0.001) demonstrating that there was agreement in the ranked order of the primary outcomes respondents considered appropriate to indicate the success of interventions for CF. Improvement in cognition was seen as the most important outcome, and an increase in physical strength as the least important (Table 7). In terms of a decision on which statements achieved consensus as important, a median cut-off of 2.98 was used.

**Table 7:** Section 10, Ranking of primary and secondary outcomes *N* = 48.

Outcome	Academic Lab scientist/biologist	Academic working with people	People with lived experience and unpaid carers	Professional carers (clinicians, social care)	Mean rank	SD
Ranking of primary outcomes *N* = 48						
**Improved cognition**	1.71	2.48	1.69	2.50	2.16	1.07
**Improved mobility**	3.29	3.09	2.54	3.00	2.96	1.10
Reduced frailty score	2.29	2.91	3.46	3.00	2.98	1.28
Reverted to non-cognitive frailty profile	3.57	2.91	4.23	2.00	3.24	1.81
Physical strength	4.14	3.61	3.08	4.50	3.65	1.32
Ranking of secondary outcomes *N* = 49						
**Extended or improved independence (e.g., assessed by Activities of Daily Living)**	2.57	3.00	2.85	3.50	2.98	1.87
**Quality of Life**	2.71	3.74	3.85	3.33	3.58	2.52
**Happiness/mental health**	5.71	3.74	2.85	5.17	3.97	2.11
**Wellbeing (e.g., including purpose in life and life satisfaction)**	4.57	5.13	5.00	4.50	4.94	3.17
**Achieving maximum potential ability**	5.43	5.65	5.31	4.50	5.39	2.74
Minimizing adverse health outcomes	6.29	5.39	5.54	5.17	5.56	2.26
Remaining productive in their careers, personal interests or family roles	4.86	6.43	5.54	6.00	5.92	2.40
Reduced social care needs	7.43	7.04	7.78	7.33	7.35	1.93
Reduced healthcare needs	7.57	7.87	7.54	5.67	7.48	2.84
Appropriate social support in place	7.86	6.91	8.69	9.50	7.84	1.87

Items where consensus was achieved according to our criteria are highlighted in bold.

The Kruskal-Wallis test examined the distribution of ranks for each outcome across the groups. There were no significant effects for any outcome except for “Revert to a non-cognitive frailty profile,” 8.29 (df = 3), *p* < 0.05. The individual group comparisons were not significant once the Bonferroni adjustment was applied.

###### Secondary outcomes

Friedman’s test showed a significant difference (Chi-Squared = 137.30, df = 9, *p* < 0.001) between the ranks. That is, there was agreement in the ranked order of the secondary outcomes that respondents thought would be appropriate to indicate success of interventions for cognitive frailty. “Extended or improved independence” was ranked as the most appropriate outcome, and whether “Appropriate social support was in place” as the least appropriate (Table 7). Reduced health and social care needs were also ranked as less important. In terms of a decision on which statements were achieving consensus as important, a cut-off rank below the median, 5.48, was used.

The independent samples Kruskal-Wallis test showed significant differences in the distribution of the ranks across the groups only for the outcomes of “happiness and mental health” [test statistic (df = 3) = 8.90, *p* < 0.05], and of “Appropriate social support is in place,” [test statistic (df = 3) = 13.09, *p* < 0.01]. The group of people with lived experience and carers ranked Happiness and Mental health as a more important outcome than did other groups, with the differences with the group of academic lab researchers being significant, *p*_adj_ < 0.05. For the outcome “Appropriate social care is in place,” the group of academics working with people judged this to be more important than did other groups, with the difference from the professional carer/clinician group being significant, *p*_adj_ = 0.01.

Participants were then asked what they thought were the main societal outcomes of interventions on cognitive frailty. As shown in Table 8, there was >70% agreement for Economic benefits and Social and Community Capital. Although agreement for “Mortality/survival time is not a useful outcome” was >70%, disagreement was also >15%. Specifically, academics working with people, and people with lived experience showed lower agreement with that statement.

**Table 8:** Section 10, agreement/disagreement on potential outcomes of interventions for society (*N* = 48).

Outcome	Academic Lab scientist/biologist	Academic working with people	People with lived experience and unpaid carers	Professional carers (clinicians, social care)	Overall % agree/disagree
**10.4 Economic benefits**	83.33	95.24	71.43	90.00	85.10%
**10.5 Societal and community capital**	83.33	95.00	92.86	80.00	91.30%
10.6 Mortality/survival time is not a useful outcome (“life to years, not years to life”)	83.33	66.67	69.23	70.00	71.7%

Items where consensus was achieved according to our criteria are highlighted in bold.

##### Section 11: effectiveness of interventions for cognitive frailty (i.e., do they work in real life?)

There was more than 70% agreement for both Questions 11.1 and 11.2, see Table 9.

**Table 9:** Section 11, agreement/disagreement on elements of effectiveness of interventions *N* = 47.

Elements of effectiveness	Academic Lab scientist/biologist	Academic working with people	People with lived experience and unpaid carers	Professional carers (clinicians, social care)	Overall % agree/disagree
**11.1 Larger sample size means more sources of variance/confounders can be assessed**	80.00	76.19	100.00	90.00	83.72%
**11.2 Determining which group of people with which characteristics may benefit from which type of intervention is important**	100.00	95.45	91.67	100.00	93.48%

Items where consensus was achieved according to our criteria are highlighted in bold.

Question 11.3 asked respondents to rank seven factors in relation to their importance in determining the effectiveness of an intervention.

Friedman’s test showed a significant difference in the ranks (Chi-Squared = 76.18, df = 6, *p* < 0.001). “Effectiveness in the real world” was ranked as the most important, and “ability to sustain the interventions for the amount of time they were needed” was seen as the least important. Three items tied for second rank (Table 10). As this included the median cut-off (3.41), the cut-off of a rank including and above the median was implemented.

**Table 10:** Section 11, Ranking of factors influencing effectiveness of interventions *N* = 48.

Factor	Academic lab scientist/biologist (*n* = 7)	Academic working with people (*n* = 23)	People with lived experience and unpaid carers (*n* = 13)	Professional carers (clinicians, social care) (*n* = 6)	Mean rank	SD
**Effectiveness (how it works in the real world)**	2.86	1.87	3.15	3.00	2.49	1.65
**Affordability and time commitment**	3.00	3.70	3.23	3.17	3.41	1.57
**Acceptability**	4.00	3.00	3.54	4.00	3.41	1.57
**Including the patient/older person voice at all stages including design**	2.29	4.13	3.38	2.00	3.41	2.24
Appropriateness (e.g., in culture)	5.71	4.48	4.77	4.59	4.74	1.58
Level of commitment from staff and older participants.	5.00	5.13	4.69	5.33	5.02	1.52
Sustaining them for the amount of time they are needed for	5.14	5.70	5.23	6.00	5.53	1.93

Items where consensus was achieved according to our criteria are highlighted in bold.

The independent samples Kruskal-Wallis test showed differences in the distribution of ranks for each intervention across the groups only for the characteristics of “Effectiveness (How it works in the real world)” [Test statistic (df = 3) = 9.18, *p* < 0.05] and “Including the patient/older person voice at all stages including design” [Test statistic (df = 3) = 7.84, *p* < 0.05]. Individual group comparisons were not statistically significant in adjusted comparisons.

##### Section 12

This section explored intervention design strategies, beginning by asking participants to respond to a set of intervention strategies as to whether they could increase the likelihood of success. There was strong agreement for all items and little variance between expertise groups, see Table 11.

**Table 11:** Section 12, Percentage agreement/disagreement with potential intervention design strategies and on overall intervention approaches.

	Academic lab scientist/biologist	Academic working with people	People with lived experience and unpaid carers	Professional carers (clinicians, social care)	% agree/disagree
**Potential Intervention design strategies.**					
**12.1 Measure effects regularly, not just at the end (to know if they work or not or when they start or stop working)**	100.00	100.00	100.00	80.00	95.7%
**12.2 Be ready to revise strategy and adjust it**	100.00	95.45	100.00	80.00	95.7%
**12.3 Engage participants and seek feedback from them regularly**	100.00	100.00	100.00	90.00	97.9%
**12.4 Ensure a maintenance component is included after the main intervention**	100.00	100.00	100.00	90.00	97.7%
**Overall intervention approaches**					
12.6 We look for a single intervention that works for most and that has multiple benefits (reduces cognitive frailty by various means)	50.00	63.64	45.00	50.00	53.3%
12.7 We go for a single target (an identified mechanism of cognitive frailty) and tackle it using a variety of interventions	33.00	59.09	63.63	40.00	53.74%
**12.8 We go for something trickier but perhaps more inclusive: aiming for several accessible combined interventions that synergize (not everyone will be able to engage in all of them, but will be able to engage in a couple that work for them) Interventions will be designed like ‘packages’ that are semi-tailored**	66.67	81.82	81.82	70.00	77.8%
**12.9 We go for a fully personalized intervention that taps into all that is possible (supported by a decisional flowchart, or by a multidisciplinary team for instance)**	50.00	86.36	66.67	80.00	78.3%

Items where consensus was achieved according to our criteria are highlighted in bold.

Respondents were then asked for their agreement or disagreement with overall intervention approaches, with 47 responding to this section. There was agreement of >70% on two of the intervention approaches, see Table 11.

### Results of Round 3

There were 38 respondents to Round 3 with only 4 people identifying as academic Lab Scientists and 4 people as Professional carers. Percentages for these subgroups should be treated with caution. There were 21 academics working with older people and 12 people with lived experience/carers. Given high levels of consensus in these analyses, tables are only presented to illustrate where there was variability in consensus across groups or for where there was no consensus. Four respondents were classified into more than one group, hence the total of 38.

#### Section 1: understanding of CF

There was strong overall consensus for these statements. Our analysis of the separate expertise groups showed that there was no consensus for Items 1.1 and 1.2 for the people with lived experience and unpaid carer group, see Table 12.

**Table 12:** Round 3, Sections 1, 2 and 5, percent agreement on items where there was not uniform consensus across groups, (*n* = 41).

1. Understanding the concept of cognitive frailty:	Academic lab scientist/biologist (*n* = 4)	Academic working with people (*n* = 21)	People with lived experience and unpaid carers (*n* = 12)	Professional carers (clinicians, social care) (*n* = 4)	Overall % agree/disagree
Understanding the concept of cognitive frailty					
1.1 Many researchers and clinicians are not clear what is meant by cognitive frailty	100%	91%	42%	100%	76%
1.2 Whilst normal aging may contribute to cognitive frailty, it is unhelpful to assume that cognitive frailty is an expected part of aging	100%	100%	58%	100%	87%
1.3 With positive attitudes and anti-discriminatory practices, resilience may improve, meaning that cognitive frailty is not inevitable	100%	91%	75%	75%	86%
Whether CF can be prevented, reversed, or onset delayed					
2.5 Examination of reversal of cognitive frailty in the literature is very rare	100%	76%	83%	50%	81%
Risk factors for cognitive frailty that could be targets for intervention					
5.4 Air pollution affects brain health especially for those with pre-existing vulnerability leading to cognitive frailty					
Developing interventions for the management of cognitive frailty					
6.1 We do not know the best timing (e.g., in terms of severity of cognitive frailty or age of person) for interventions to enable reversal	100%	67%	63%	50%	67.38%

#### Section 2: whether CF can be prevented, reversed, or onset delayed

There was clear overall consensus for every item in this section. However, there was no consensus amongst Professional carers/clinicians regarding Item 2.5 on the rarity of examinations of reversal of CF in the literature, where there was only 50% agreement, see Table 12.

There was overall consensus and also consensus within each of the groups for the following sections:

- Section 3, Screening for CF;
- Section 4. Possible mechanisms for interventions to address CF.

#### Section 5: acknowledged risk factors for cognitive frailty that could be targets for interventions

There was overall agreement as well as agreement within groups for all items in this section, except Item 5.4 regarding air pollution, where there was no consensus within the group of people with lived experience and carers (only 43% agreement), see Table 12.

#### Section 6: developing interventions for the management of cognitive frailty

All statements showed consensus except for 6.1 “We do not know the best timing (e.g., in terms of severity of cognitive frailty or age of person) for interventions to enable reversal” where there was no consensus by our 70% criterion for any subgroup except the academic lab-scientists (mean agreement was 67.38%), see Table 12.

For the remaining sections, all items achieved consensus for all statements overall and within groups. These sections are listed as follows:

- Section 7: Important targets for interventions for cognitive frailty to focus on,
- Section 8: Possible restrictions on for whom interventions for cognitive frailty might be suitable,
- Section 9: Factors that may restrict or affect the feasibility or likelihood of adhering to interventions for cognitive frailty,
- Section 10: Attention to the impact of inequalities and person variables on the feasibility of different types of interventions
- Section 11: Primary outcomes of interventions for Cognitive Frailty that we should target,
- Section 12: Secondary outcomes of interventions we should target,
- Section 13: Outcomes of interventions on cognitive frailty for society,
- Section 14: Importance in determining the effectiveness of an intervention,
- Section 15: Intervention design strategies to increase the likelihood of success,
- Section16: Further factors in intervention design.

In summary, there was consensus on 89 out of 90 statements overall, with only four items showing a subgroup without consensus. In the only item that did not show overall consensus “We do not know the best timing (e.g., in terms of severity of CF or age of person) for interventions to enable reversal,” there was also a lack of consensus with the statement for three of the four subgroups, with only academic lab scientists showing consensus agreement with the item.

## Discussion

With 9% of the over 60s population estimated to be living with cognitive frailty (Qiu et al., 2022), cognitive frailty is a critical global concern associated with aging. Given the increasing number of older people globally, an increased number of people living with CF is likely, as is the associated risk of disability resulting from this co-existence of physical and cognitive impairments. CF is a potentially preventable risk state for later life consequences such as dementia and increased need for health and social care support and an important target to reduce the gap between healthy lifespan and actual lifespan, that is, healthy aging. It is also a pressing concern given the wide variation in healthcare utilization and opportunities to access and use clinical and rehabilitation services to identify and treat frailty and cognitive decline. An increased awareness of the importance of prevention and building resilience is needed to support mid-life populations to promote or maintain healthy aging in their communities.

This Delphi consensus study set out to explore and develop consensus around interventions for CF among experts across disciplines and sectors, including people with lived experience of CF, and those providing care. Views of those in carer roles, unpaid or as part of their employment, were important to include, in relation to their understanding of CF and interventions for it, recognizing sub-clinical functional effects of CF. Given that previous resources and reviews on interventions for CF had largely been from the point of view of applied health researchers or clinicians, this study represents a first attempt to obtain agreement across a broader spectrum of involved experts on critical factors for interventions. The study brought in a broader mix to include the perspectives of people who may be involved in the decisions about which interventions to operationalize with specific patients or population groups, the potential end users and those who care for them, as well as basic laboratory scientists whose perspectives may not often be considered in relation to operationalization or methods of health interventions and who are not often involved in such operationalization or design. These results emphasize the need for tailored interventions that consider both individual and structural determinants of cognitive frailty. The study’s interdisciplinary consensus approach suggests potential for broader collaboration in future frailty intervention frameworks, incorporating biological, social, and policy-driven perspectives.

Delphi studies by their design are iterative. Out of the original 127 items presented to participants in Round 2, there was consensus on 74, and although fuller consensus was achieved in the final round (Round 3), information can also be gathered from some of the items where consensus was not achieved in Round 2. Little consensus was observed in the section on understanding of the concept of CF. Notably, there was a lack of consensus on whether it was a help or a hindrance to normalize CF as an expected part of aging. For some, there is therefore a belief that CF could be a normal part of aging. While physiological changes that occur with increasing age are clearly a risk factor for CF, our own work and that of others (see Holland et al., 2024 for a review) have identified risk factors that may worsen, and in contrast, factors that reduce, the rate at which age-related biological changes occur. Examples are factors that impact on immune system aging such as stress or diet, or impacts of healthy lifestyle on cardiovascular health. Increasing understanding that some age-related conditions can be prevented, delayed or reversed is a crucial step toward countering beliefs about inevitability. That is, although reviewed evidence shows increase in likelihood of CF with increasing age, evidence also describes it as a distinct, non-universal and mainly pre-clinical condition associated with these risk factors. Nevertheless, given that the respondent group included people with lived experience, we also need to acknowledge that some people were in a situation where accepting the impacts of aging was an important part of their own coping and resilience.

There was, however, consensus on “Negative attitudes toward aging are what need changing” suggesting that experts reflected on the negative stereotypes associated with old age (including ageism) and considered that CF might be seen as a low priority for interventions. This a known problem with significant ‘self-stigma’ and wider societal stigma associating any changes in old age with commonly held beliefs that cognitive decline and frailty are inevitable conditions of older age (Preville et al., 2015). Linking to the statement on normalizing CF as a normal part of aging being seen as a hindrance, this may be because of the negative attitudes toward aging in general and the consequences this has, i.e., not prioritizing intervention for CF. Given the accurate identification by the World Health Organisation of ageism as one of the four key pillars of the Decade of Healthy Aging (World Heath Organisation, 2021), it is essential to challenge even “compassionate ageism” which incorporates stereotypical assumptions that frame mindsets, behaviors and clinical interventions, almost making frailty and cognitive decline a self-fulfilling prophecy. Providing strong evidence to support a more positive aging agenda is crucial and so emphasis on preventative interventions is critical.

The laboratory-based scientists’ level of consensus diverged from views of those with lived experience and clinicians on some topics, highlighting that the biological science associated with frailty and brain health at a mechanistic level needs to be factored into the consideration of interventions. The interaction between the potential for biological mechanisms and lifestyle, environment or policy interventions is explored further in our scoping reviews (Fowler Davis et al., 2024a; Fowler Davis et al., 2024b; Hodgson et al., 2024; Holland et al., 2024), and this Delphi study suggests that understanding and knowledge exchange across the disciplines could enhance potential theory-based design of interventions. Likewise, the high consensus associated with “Many researchers and clinicians are not clear what is meant by cognitive frailty” suggests that more needs to be done to explain and differentiate CF from purely brain related ill-health including dementias, while still accepting that people with different expertise will bring different perspectives to the mix.

Ranked items showed that respondents clearly differentiated between items such as intervention targets, factors affecting feasibility of interventions including structural factors, the impact of personal circumstances, and primary and secondary outcomes, as well as factors that may influence intervention effectiveness, such as accessibility, affordability, acceptability and addressing health literacy.

There was broad agreement on intervention design strategies. However, there was no consensus on simpler interventions–looking for a single intervention that works for most people, or a single target mechanism, and instead there was consensus on inclusive, combined and personalized interventions, e.g., that may involve a multidisciplinary team, underlining the understanding that a broad view of the factors influencing CF will link to a broad view of interventions.

Consensus shown for screening, bespoke assessment and specialist care needs to be carefully considered against the current demand for memory services. Development of early biomarkers and early screening for people at high risk were suggested strategies. Alternative methods to enable self-monitoring and self-management may be indicated, for example, with digital tools and online assessments, perhaps using Artificial Intelligence facilities.

Differences between the groups of experts were examined and a notable finding was that there were fewer differences than one might expect. However, it is important to note some of the places where there were differences; for example, in Round 3, there was no consensus among respondents with lived experience and carers in terms of items “Many researchers and clinicians are not clear what is meant by cognitive frailty” and “Whilst normal aging may contribute to CF, it is unhelpful to assume that cognitive frailty is an expected part of aging.” The lack of consensus on the latter statement can be attributed to the conflict between knowledge that certain deficits increase in likelihood with increasing age, versus knowledge that age, per se, does not cause them, rather, that a range of mechanisms that are subject to a range of risk factors and environmental influences affect the variability observed within the older population. A further difference where this group did not show consensus, but the others did, was the item referring to the impact of air pollution on brain health, especially for those with pre-existing vulnerability, leading to cognitive frailty. This is also evidenced in the literature (e.g., Power et al., 2016; Hodgson et al., 2024), but may well be an example of more recent evidence that is relatively well-known amongst researchers but not yet in the public domain.

Other differences between expert groups showed that the Lab scientists (who were all biologists), showed differential consensus on specific items in Round 2. For example, “The role of the individual is important, rather than just a focus on biological or environmental mechanisms” showed no consensus for the Lab scientists but consensus for the other groups, and “Intervening on more direct biological mechanisms may be quicker” showed consensus for the academic lab-scientists but not for any other group. Bringing together researchers and other experts who emphasize the individual person together with biologists who put more emphasis on the underlying biological mechanism in development of interventions is an important exercise as we seek to put all our expertise together to solve the challenges of aging and have impact beyond what we can at present; this is illustrated by these differences and is a strength of this approach.

The influence of people’s differing experiences was reflected well in Section 7 on “Possible restrictions on for whom interventions for cognitive frailty might be suitable.” This suggests the importance of discussing the difficulties groups experience when implementing interventions at the point of design, including acknowledging the different roles the different groups play in implementation and reception of interventions.

Finally, there was no consensus overall that CF can be reversed, with experts by experience showing the highest disagreement with this concept, although there was consensus for the other groups. Given that there is evidence (although limited as yet for reversibility in the literature; e.g., Ruan et al., 2020), this suggests that more work is needed to engage people with lived experience to promote this possibility, given that if there is little expectation of improvement once CF is present, motivation for intervention may be lower. However, further investigation is needed to understand this group’s perspective, ensuring people are distinguishing between dementia and the cognitive decline associated with CF. The consensus on the statement that few intervention studies directly address the reversibility question seems to tally with literature searches and suggests that this is an area where further research is needed. Many interventions have shown reductions in frailty and cognitive impairment (for a recent review, see Ji et al., 2025), but studies on reversibility are not absent, e.g., Huang et al. (2025) demonstrated reversal from Reversible CF (RCF) to non-cognitively frail (non-CF) and from potentially reversible CF (PRCF) status to both RCF and non-CF status in multi-domain interventions. Although statistically significant, the proportion of participants reversing was still small and further research is needed to determine why some people show reversing and others do not. A further study again using a multi-domain intervention (WE-RISE ™), found that after 12 weeks, 74% of the intervention group were no longer assessed as having cognitive frailty whereas only 10.7% of the control group were no longer cognitively frail (Murukesu et al., 2024). Further research in different populations with carefully identified RCF and PRCF groupings would undoubtedly improve the understanding of the likelihood of reversal across the expert groups. Nevertheless, there is clear evidence that reversal to a non-CF state can result from intervention.

### A way forward

Although it should be emphasized that this study is based on expert consensus, rather than direct testing of interventions, many of the academic experts did refer to supporting evidence in the open text boxes. One way to consider next steps to developing interventions is suggested by Wight et al. (2016), who suggest six steps for quality intervention development (6SQuID). The first few steps align with our progress in this consensus development and the evidence syntheses that preceded it, as we have sought to (1) define and understand the problem and its causes; (2) identify which causal or contextual factors are modifiable including which have the greatest scope for change and who would benefit most; and (3) deciding on the mechanisms of change. The latter three steps of Wight et al. provide suggestions as to what is next which should be considered in further development following this consensus stage: (4) clarifying how interventions will be delivered; (5) testing and adapting the intervention; and (6) collecting sufficient evidence of effectiveness to proceed to a rigorous evaluation.

1. Define and understand the problem and its causes

The primary outcomes of any planned intervention need to address the outcomes identified via the consensus such as cognition and mobility, ensuring understanding of definitions of CF (and so inclusion in interventions). Secondarily, older adults with CF will experience a need for improved independence, quality of life and wellbeing. These broad interventions should be affordable, timely and meaningful (meet personal attitudes and beliefs. About the effectiveness of an intervention). Mobility, affordability, access to screening methods and physically accessible environments and healthy foods were all deemed significant to the cause and effects of CF.

1. Identify which causal or contextual factors are modifiable including which have the greatest scope for change and who would benefit most

There was a strong consensus that CF can be prevented, delayed or reduced, given knowledge on risk factors. Biological mechanisms could be targeted alongside social and psychological factors that involve long-term changes to lifestyle, and these may include environmental exposure. In this respect policy changes are needed to influence psychosocial/socioeconomic factors that disproportionally place some older adults at risk of CF.

Delaying CF was viewed as dependent on personal behavior but also on genetic factors. Risk factors may be individual but also interact in complex ways so require multiple interventions. Early assessment/screening of older people would help identify factors for earlier interventions, rather than only identifying a problem when the individual encounters health care practitioners.

Overall, there is a belief among some respondents that CF might not be reversible (and the best timing for interventions to enable reversal is not known, possibly because there is limited evidence for reversal in the literature), but it could be improved, although evidence on reversibility is building. Interdisciplinary working to test interventions in different models and systems (including cell culture and animal models from invertebrate to vertebrate) will help to improve likelihood that pre-clinical studies are clinically relevant.

Experts agreed that effectiveness (how it works in the real world), affordability and acceptability (for example, in terms of time commitment), were important and that interventions should be planned together with the affected person. Intervention acceptability would be based on the early measurement of effect with the option to revise and adjust interventions and to ensure that a maintenance component is included.

1. Deciding on the mechanisms of change.

Interventions need to be personally relevant (e.g., acceptable in different cultures) and personalized. Biological or environmental mechanisms may vary, and so intervention goals should target lifestyles to protect individuals from decline. Healthy behaviors including physical activity might be helpful but a focus on multi-domain intervention and common mechanisms such as psychosocial and socioeconomic factors should be emphasized. For those with urgent needs, a fast-acting personalized intervention should be considered but long-term lifestyle modification may also be suitable for different people. Physical activity with diet and probiotics, targeting isolation and loneliness, and strength-related exercise, are all important although with recognition that other morbidities such as arthritis will influence individual ability to complete those. There was consensus that agency and involvement of the person in agreement on choice of interventions was vital. There was also agreement that screening was essential, particularly that high-risk people should first be screened at midlife.

The implication of the consensus was that a multi-modal, co-produced health promotion approach will be needed, and that guidance should be used to address how to develop screening and assessment interventions alongside treatment (O'Cathain et al., 2019). This is consistent with several trials that demonstrate the effectiveness of multi-domain interventions often delivered as complex interventions for cognitive and physical frailty (Ng et al., 2015; Dedeyne et al., 2017) and for reversion of CF status (Murukesu et al., 2024).

The reversibility of CF is an extremely important differentiating factor for investment in interventions and an outcome measure suggesting treatment effectiveness will be critical for any future trial and research programs. Respondents agreed that improved cognition, improved mobility and reversal to a non-cognitive frailty profile would be the important outcomes to show effectiveness.

The dominant narrative within the section on “Approaches to interventions for cognitive frailty” suggests that most experts share values around person-centered care and assessment of wellbeing, widely agreeing upon these as critically important. However, this presents a significant challenge to researchers, given that the predicted incidence of CF may be as high as 9% of the older population (Qiu et al., 2022) and a population health approach is needed for both treatment and prevention. Although the role of person-centered or culturally specified interventions was agreed upon as necessary (“one size does not fit all”), there is a need for theory-driven, generalizable approaches that can enhance efficiency, cost-effectiveness, and scalability of existing implementation approaches (Schleider and Beidas, 2022) for CF. Scalable but contextualized interventions seem the way forward.

### Limitations and strengths

Self-selection of experts is a strength of this study, particularly the inclusion of those with lived experience. However, self-selection across a range of disciplines in the CF network is a novel approach, that necessitated a disaggregation of the responses of separate groups, to examine any differences. The number of respondents in total was seen as comparable to other previous Delphi studies (e.g., Sezgin et al., 2022, had 21 experts), although the small number in some groups in Round 3 (notably laboratory scientists and professional carers/clinicians) is a limitation. The participant group was diverse in terms of geographical spread, including experts from UK, Europe, North and South America, and Asia. From our examinations of the diversity within the CF Interdisciplinary network (CFIN) in which this study was based, we know that we have good diversity of ethnicities. However, despite significant Chinese representation of CF expertise in the literature, we did not have any respondents for that country, and so this could be a limitation. Nevertheless, the approach provides broader face validity to the consensus achieved (e.g., see Nair et al., 2011). We intend that such common shared understanding can underline the roles of all these groups in development of interventions or population-level policy changes to impact the mitigation of CF and healthcare utilization, with an emphasis on prevention of disability, and associated need for care support.

Given the focus on interventions, there was no exploration of understanding of the aetiology of CF, and causes of CF were not included in the Delphi. Understanding of mechanisms will be needed to suggest how interventions would target and potentially reverse the progress of the syndrome. Nevertheless, a strength of this study is the clear consensus on lifestyle and behavioral interventions concurring with various previous reviews indicating that the syndrome is affected by personal behavior change. Our previous scoping reviews of mechanisms also suggest that behavior change may be one important factor that links underlying mechanisms to both healthy aging and also outcome CF (although there are other linking variables as well, including depression and anxiety, Holland et al., 2024) and environmental issues (Fowler Davis et al., 2024a; Fowler Davis et al., 2024b; Hodgson et al., 2024).

The lack of consensus on biologically based interventions also suggested that our experts were more interested in developing broad lifestyle intervention than a single biologically based drug therapy. None-the-less, given the dominance of pharmacological solutions and therapeutic methods, there may be existing or newer pharmacological or nutritional interventions worthy of further investigation for managing brain health, for example Vitamin D as a neuron therapeutic for reducing inflammation (Murukesu and Manucha, 2024).

We did not collect demographic information such as age and ethnicity, although we know that the CFIN network on which this study was based is relatively diverse. However, future work could usefully explore how cultural factors influence both the acceptability and effectiveness of CF interventions.

The value of the input of people with different expertise is demonstrated by this study> Although many of the proposed recommendations were based on existing evidence as well as the consensus achieved in this study, they still require validation through experimental studies.

## Conclusion

The consensus achieved in this study associated with CF interventions needs to be considered as a first step in defining health promotion activities and interventions. The consensus provides a foundation for policy-guided, preventive approaches. Given the prevalence and resultant potential disability in older adult populations, the consensus statements are important and represent expert opinion that is inter-sectoral. Research in this direction will further inform public health policies to implement evidence-based research findings to the development of prevention plans (Kelaiditi et al., 2013). However, given what we know about the length of the gap between what academic researchers know and actual implementation in healthcare or policy changes (Morris et al., 2011), or adoption by patients, a new way of planning and promoting the need for prevention and intervention in this area is needed. This study takes a crucial step toward changing current siloed approaches, including all stakeholders from the outset. Interdisciplinary involvement of the broad groups identified here needs further development in future work.

## Data Availability

The anonymised data supporting the conclusions of this article will be made available by the authors, without undue reservation.

## Footnotes

^1^**VOICE** is a community of public, patients and carers. Embedded in the UK National Innovation Centre for Ageing at Newcastle University, it supports public and patient involvement and engagement (PPIE) in research.
